# Intermittent vibrations accelerate fracture healing in sheep^1^

**DOI:** 10.1590/s0102-865020190070000002

**Published:** 2019-09-12

**Authors:** Degong Mu, Jing Yu, Junhao Lin, Chen Li, Baohui Hao, Feng Gu, Chao Liu, Lei Tan, Dong Zhu, Xizheng Zhang

**Affiliations:** I Bachelor, Operating Theatre 3, the First Hospital of Jilin University, Changchun, China. Conception and design of the study, analysis and interpretation of data, manuscript writing.; II Master, Operating Theatre 1, the First Hospital of Jilin University, Changchun, China. Manuscript writing; III Master, College of Mechanical Science and Engineering, Jilin University, Changchun, China. Statistics analysis.; IV Master, Department of Orthopedic Traumatology, the First Hospital of Jilin University, Changchun, China. Technical procedures.; V Master, Department of Orthopedic Traumatology, the First Hospital of Jilin University, Changchun, China. Technical procedures, interpretation of data, critical revision.; VI Master, Department of Orthopedic Traumatology, the First Hospital of Jilin University, Changchun, China. Technical procedures, interpretation of data.; VII Master, Department of Orthopedic Traumatology, the First Hospital of Jilin University, Changchun, China. Statistics analysis.; VIII PhD, Department of Orthopedic Traumatology, the First Hospital of Jilin University, Changchun, China. Analysis and interpretation of data, critical revision.; IX PhD, Department of Orthopedic Traumatology, the First Hospital of Jilin University, Changchun, China. Conception and design of the study, analysis and interpretation of data, critical revision.; X PhD, Institute of Medical Equipment, Academy of Military Medical Sciences, Tianjin, China. Critical revision.

**Keywords:** Vibration, Fracture Healing, Finite Element Analysis, Sheep

## Abstract

**Purpose:**

To investigate the effect of intermittent vibration at different intervals on bone fracture healing and optimize the vibration interval.

**Methods:**

Ninety sheep were randomized to receive no treatment (the control group), incision only (the sham control group), internal fixation with or without metatarsal fracture (the internal fixation group), and continuous vibration in addition to internal fixation of metatarsal fracture, or intermittent vibration at 1, 2, 3, 5, 7 and 17-day interval in addition to internal fixation of metatarsal fracture (the vibration group). Vibration was done at frequency F=35 Hz, acceleration a=0.25g, 15 min each time 2 weeks after bone fracture. Bone healing was evaluated by micro-CT scan, bone microstructure and mechanical compression of finite element simulation.

**Results:**

Intermittent vibration at 7-day interval significantly improved bone fracture healing grade. However, no significant changes on microstructure parameters and mechanical properties were observed among sheep receiving vibration at different intervals.

**Conclusions:**

Clinical healing effects should be the top concern. Quantitative analyses of bone microstructure and of finite element mechanics on the process of fracture healing need to be further investigated.

## Introduction

Fracture healing is a complex process that involves the coordination of a sequence of biological events, and is delayed in approximately 5-10% of bone fractures due to various causes^1^ ^,^ ^2^. Efforts have been made to shorten healing time, and improve healing quality. During t bone development and growth, the size, shape and intensity of the bone largely depend on mechanical stimulation, the responses to which could promote bone modeling and remodeling^3^.

Low-load mechanical vibration has been reported to increase bone mass, promote bone growth, and enhance fracture healing. Furthermore, mechanical vibration significantly increased torsional stiffness and energy absorption of rabbit tibial bone during fracture healing, and improved callus formation and other mechanical features of fracture healing in sheep tibial bone^4^ ^–^ ^7^. Tibial fractures in patients healed faster with axial vibration than those without^8^.

In addition, evidence suggests that dynamic stress more effectively sensitizes bone cells to stimulation than static stress, and intermittent vibration is superior to sustained vibration in promoting bone growth^9^ ^,^ ^10^. The intervals of intermittent vibrations help to restore the sensitivity of osteoblasts to mechanical stimulation. Recent studies have shown that the levels of osteogenesis-associated proteins, including BMP-2, p-ERK, Runx2 and OCN, were upregulated by vibration in ovariectomized rats, and increased vibration time resulted in better bone formation^11^ ^–^ ^13^.

Our previous studies have shown that intermittent vibration works better than continuous vibration on bone growth against osteoporosis in terms of improving tissue biomechanical, morphometric and molecular components^14^ ^,^ ^15^. However, the optimal cycle of intermittent vibration for fracture healing has not been defined and still remains to be further investigated. In this study, we evaluated the effects of intermittent vibration on fracture healing by micro-CT and carried out finite element analysis to explore the optimal cycles with various vibration intermittences for fracture healing.

## Methods

The study protocol was approved by the local ethics committee at the authors’ affiliated institutions. All experiment procedures were carried out in accordance with the Regulatory Guideline on the Use of Experimental Animals (China, 2011). Ninety healthy 1-year-old shorttailed Han sheep (Jilin Animal Experimental Center, Jilin, China) were housed in the same environment including lighting, ventilation, humidity, and temperature, with *ad libitum* access to sunshine, water and food. The sheepcotes were regularly cleaned up to ensure a good feeding environment.

### Sheep metatarsal fracture model

The metatarsal fracture of the right hind limb of sheep was generated by osteotomy. Briefly, dimethylaniline hydrochloride, 0.6 mg/kg was injected peritoneally for anesthesia. Then, the right hind limb was shaved and disinfected with iodophor. A 12-cm incision was cut at the middle of the metatarsal along the axis of limb, the metatarsal and peripheral soft tissue was blunt separated, and the middle of the metatarsal was exposed. The limited-contact dynamic compression plate (LC-DCP) was then fixed to the metatarsal with screws, and anatomical standard osteotomy was performed using a wire saw (fracture line width 1 mm). Then, the surgical area was rinsed with saline and coated with penicillin. The incision of soft tissue was stitched with the antibacterial VICRYL* suture. Finally, the wound was disinfected with iodophor and wrapped with gauze dressing. The sheep were allowed for free postoperative movement. Antibiotics were given by intramuscular injection at the first three days for preventing wound infection.

All animals were sacrificed at the 10^th^ week after fracture was created. The metatarsals of both hind limbs of each sheep were separated from muscle and connective tissue immediately after sacrifice, and the internal fixation plate was removed. The metatarsals were immersed in 10% formalin solution. After embedding, all samples were kept at -80°C.

### Treatments

The sheep were randomized to receive no treatment (the control group), incision only (the sham control group), internal fixation with or without metatarsal fracture (the internal fixation group), and continuous vibration in addition to internal fixation of metatarsal fracture, or intermittent vibration at 1, 2, 3, 5, 7 and 17-day interval in addition to internal fixation of metatarsal fracture (the vibration group). Vibration was done 2 weeks after surgery with F = 35 Hz, and a = 0.25 g^14^ using a self-designed large animal fixation platform. The hind limb was fixed on the top of the platform. Vibration was applied vertically to the leg. An accelerometer was used to measure the acceleration. Vibration lasted for 15 min in each treatment session, and the entire vibration treatment lasted for 8 weeks.

### Micro-CT scan

**Figure 1:**
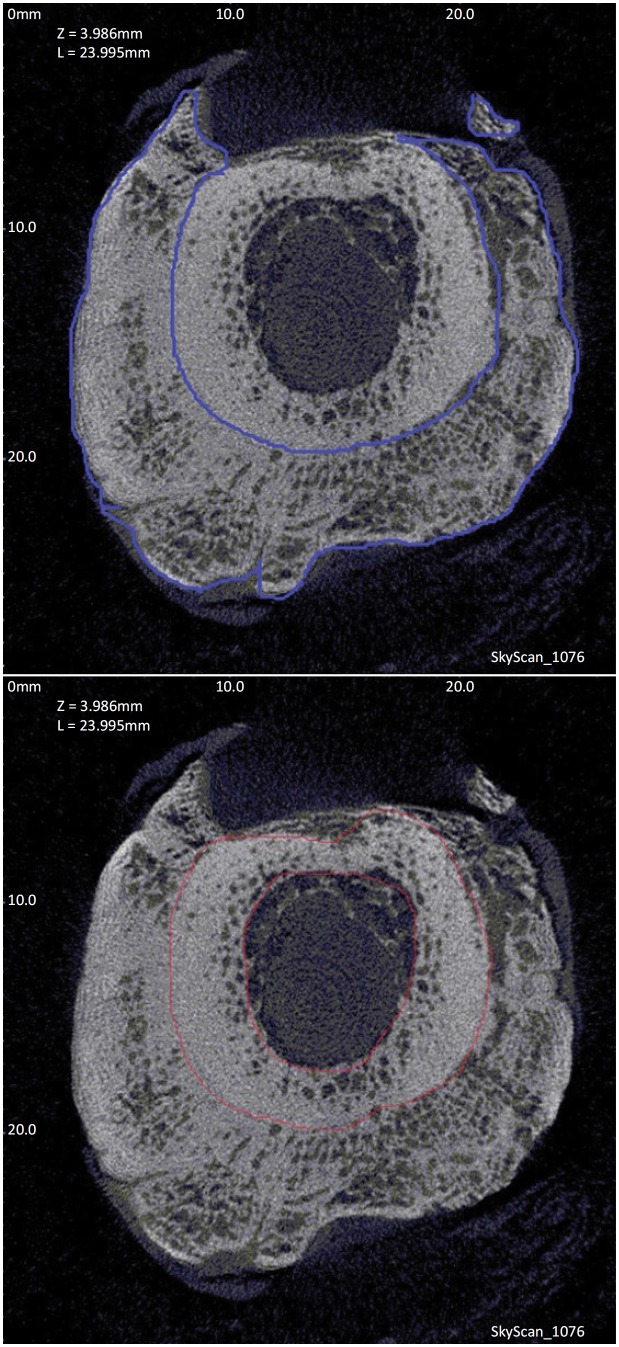
Region of interest (ROI) delineated with CTAn. Left: the callus section is shown between blue lines; Right: the cortical bone region is shown between red lines.

Micro-CT scan was performed by a radiologist who was blind to the study data. The scallops were placed in the center of the scanning area of micro-CT (Skyscan 1076, Skyscan, Belgium). Continuous bone scan (scanning voltage 70 kv, current 142 A, filter Al 1.0 mm, graphic resolution of 38 mm) was performed along the long axis of the metatarsals and centered on the fracture line. Three-dimensional (3D) reconstruction of original micro-CT images was carried out to observe fracture healing and 3D microstructure. Briefly, original scan images containing the mass values and the cross section geometry of internal and external structure of the measured objects were converted into the sectional view (bmp format) by NRecon software. The bmp format sectional views were imported into the CTAn software (with Skyscan1076 software package), and the regions of interest (ROI) were delineated. The internal and external callus was selected within the cross section of the reconstructed 3D micro-CT images to define ROI. Then, the ROI was scanned layer by layer using the CTAn software (Fig. 1), and the -3D microscopic parameters of bone scab were calculated. The abnormal values and the necrotic bone data of the fractures were excluded. The mean values of the 3D microstructural parameters of each group were calculated.

Then, the following 3D microstructure values were analyzed, including bone mineral density (BMD), bone volume fraction (BV/TV), trabecular bone thickness (Tb-Th), number of trabecular bone (Tb-N), and bone trabecular separation (Tb-Sp).

The quality of fracture healing is closely related to the clarity degree of the fracture line and the number of callus. Clinically, the evaluation of the fracture healing is mainly based on the clarity of the fracture line and the distribution of the callus by X-ray. Since the reconstructed two-dimensional image of the micro-CT scan image is similar to the X-ray film, we can evaluate micro-CT images similarly. To this end, the orthopedists from Bethune First Hospital of Jilin University determined the quality of bone healing in each group. The fracture healing grade was classified according to the clarity of the fracture line and the distribution of callus. Grade 0: fracture line is clearly visible without callus; Grade 1: fracture line is visible with the callus formation over the cortex; Grade 2: the fracture line is not clear with a large amount of callus bridge; Grade 3: the fracture line disappeared with the complete callus bridge.

### Finite element analysis

**Figure 2:**
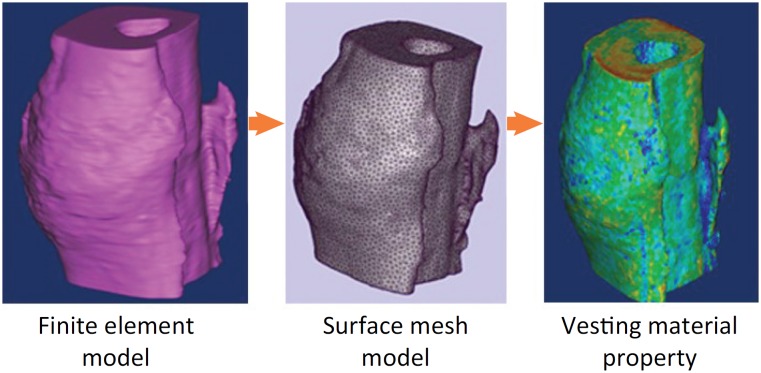
The three-dimensional model of finite element simulation.

The 3D micro-CT images were transformed into a finite element model for distribution of material properties and microscopic finite element analysis^16^. Firstly, a 3D model was established (Fig. 2) with the Mimics software, and introduced into Magics software to divide the surface mesh optimization. Then, the surface mesh was converted into the volume grid by ABAQUS software, and the material properties were assigned finally in Mimics. The computational model of macroscopic compressive elastic module is commonly used for evaluating microscopic mechanical properties^17^ ^,^ ^18^. Through the finite element simulation of loading compression experiments, the inverse resistance and elastic modulus can be calculated by the known strain values. The equation is as follows:

E=σ/ε(3−1)

Where e represents average strain and s stands for compressive stress; both are derived from the following equation, respectively:

ε=Δ1/H(3−2)

σ=F/S(3−3)

Where H is the sample height, the amount of compression deformation, and F is the compressive stress (calculated by finite element), and S is the average area of the upper and lower surfaces of the model (measured by micro-CT analysis software). In this experiment, e was 5%, that is, 0.05, elastic modulus E can be derived from the formula (3-1). In this study, we only measured the compressive elastic module of the internal fixation group and the vibration group, but not the control group and the sham control group. The reason is that there is no steel plate and stress occlusion effect in the control group and the sham control group, compared with other groups.

### Statistical analysis

Data were analyzed using SAS9.3 software. All measurable data were expressed as mean ± standard deviation (SD). Comparisons among multiple groups were analyzed with ANOVA with post hoc of Student-Newman-Keuls Test, when the difference was statistically significant. The frequency data were analyzed using the rank and Chi-square test. All tests were two sided. *P* <0.05 was considered as a significant difference.

## Results

### Grades of bone healing in sheep

During operation, one sheep died of anesthesia. Ten sheep had wound infection, which was managed by debridement and received antibiotics till the infection was controlled. However, one from the natural healing group died due to severe infection before the end of the experiment. There was no significant difference in fracture healing levels among different groups (Table 1). Nevertheless, 71.4% of sheep receiving intermittent vibration at a 7-day interval and 57.1% of sheep receiving intermittent vibration at a 5-day interval achieved grade 3 bone healing while only 13.4% of the sheep undergoing natural healing had grade 3 bone healing. In addition, 42.9% each of sheep receiving continuous vibration achieved grade 2 and 3 bone healing.

**Table 1:** The comparisons of fracture healing grade among groups.

Groups	n	Healing grade, n (%)			P
		Grade 0-1	Grade 2	Grade 3	
NH	7	1 (14.3)	5 (71.4)	1 (14.3)	0.776
CV	7	1 (14.3)	3 (42.9)	3 (42.9)	
IV-1	7	1 (14.3)	4 (57.1)	2 (28.6)	
IV-3	6	1 (16.7)	3 (50.0)	2 (33.3)	
IV-5	7	1 (14.3)	2 (28.6)	4 (57.1)	
IV-7	7	1 (14.3)	1(14.3)	5 (71.4)	
IV-14	7	1 (14.3)	4 (57.1)	2 (28.6)	

### Intermittent vibration improves bone microstructure

**Figure 3:**
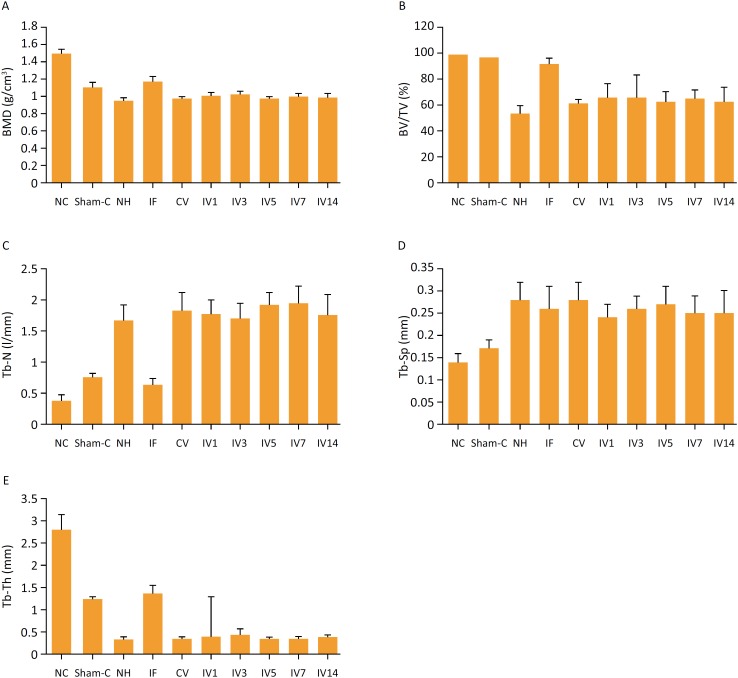
Comparisons of different evaluation parameters among 10 groups were quantitatively measured with micro-CT.

BMD, BV/TV, Tb-Th, Tb-N and Tb-Sp were evaluated with the 3D microstructure in this study (Table 2, Fig. 3).

**Table 2:** The multiple comparisons of three-dimensional microstructure evaluation among groups.

Evaluated Parameters	NC	Sham-C	NH	IF	CV	IV1	IV3	IV5	IV7	IV14	P*
BMD (g/cm^3^)	1.49 ± 0.05^a^	1.10 ± 0.06^c^	0.94 ± 0.04^e^	1.17 ± 0.06^a^	0.97 ± 0.02^ed^	1.00 ± 0.04^ed^	1.02 ± 0.03^ed^	0.97 ± 0.02^ed^	0.99 ± 0.04^ed^	0.98 ± 0.05^ed^	<0.001
BV/TV (%)	98.67 ± 0.18^a^	96.04 ± 0.19^a^	52.42 ± 7.28^b^	90.93 ± 4.67^a^	61.07 ± 3.01^b^	64.93 ± 11.62^b^	65.56 ± 17.52^b^	61.81 ± 8.10^b^	64.26 ± 7.05^b^	61.89 ± 11.57^b^	<0.001
Tb-N (1/mm)	0.37 ± 0.11^a^	0.76 ± 0.06^b^	1.66 ± 0.26^c^	0.63 ± 0.11^ab^	1.82 ± 0.30^c^	1.77 ± 0.23^c^	1.70 ± 0.25^c^	1.92 ± 0.20^c^	1.94 ± 0.28^c^	1.75 ± 0.34^c^	<0.001
Tb-Sp (mm)	0.14 ± 0.02^a^	0.17 ± 0.02^a^	0.28 ± 0.04^b^	0.26 ± 0.05b	0.28 ± 0.04b	0.24 ± 0.03^b^	0.26 ± 0.03^b^	0.27 ± 0.04^b^	0.25 ± 0.04^b^	0.25 ± 0.05^b^	<0.001
Tb-Th (mm)	2.78 ± 0.35^a^	1.23 ± 0.05^b^	0.31 ± 0.06^c^	1.35 ± 0.19^b^	0.32 ± 0.05^c^	0.37 ± 0.92^c^	0.41 ± 0.15^c^	0.33 ± 0.03^c^	0.33 ± 0.04^c^	0.36 ± 0.07^c^	<0.001

* indicating the P value between multiple groups. The different upper letters indicate P<0.05 between two groups, and the same upper letters indicate P>0.05 between two groups.

BMD, BV/TV, and Tb-Th were higher in the control and sham control group and the internal fixation group than other groups (Fig. 3A, B and E). However, Tb-N was very low in the control and sham control group and the internal fixation group (all *P* < 0.05, Table 2, Fig. 3C). More importantly, Tb-N and Tb-Sp were higher in the vibration group compared with the control and sham control group and the internal fixation group (Fig. 3C-D). BMD, BV/TV, Tb-N, Tb-Sp and Tb-Th did not show any statistical difference among sheep receiving vibration at different intervals (Fig. 3A-E).

### Comparisons of E values of elastic modulus among groups

**Figure 4:**
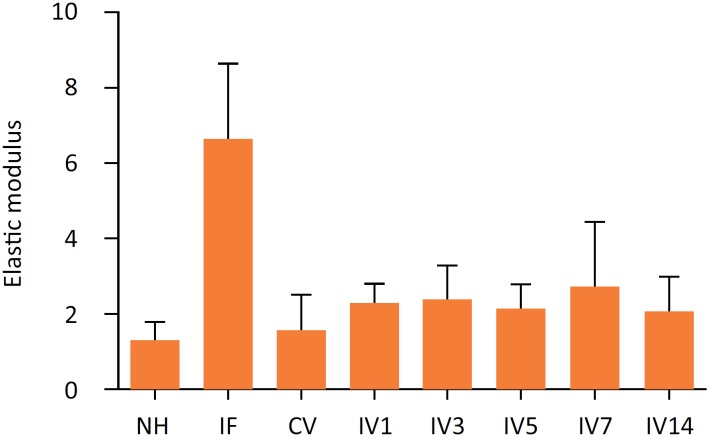
E values of the elastic modulus.

The E value of elastic modulus in the internal fixation group was higher than all other groups (*P* <0.001) (Table 3, Fig. 4). Among sheep with metatarsal fracture, the E value of elastic modulus of sheep with natural healing was only lower than that of sheep receiving intermittent vibration at 7-day interval (*P*<0.05), suggesting that intermittent vibration at 7-day interval had the best enhancing effect on fracture healing. The E values were similar among sheep receiving intermittent vibration at other intervals.

**Table 3:** The E values of elastic modulus.

	NH	IF	CV	IV1	IV3	IV5	IV7	IV14	p*
Elastic modulus	1.27±0.49^a^	6.64±1.98^b^	1.58±0.89^a^	2.26±0.49^a^	2.37±0.87^a^	2.12±0.66^a^	2.69±1.7^a^	2.08±0.88^a^	<0.001

* indicating the P value between multiple groups. The different upper letters indicate P<0.05 between two groups, and the same upper letters indicate P>0.05 between two groups.

## Discussion

Based on our results, to compare with natural fracture healing, we first found that low load mechanical vibration can improve fracture healing. Intermittent vibration effect on healing is better than continuous vibration effect, the best effect of 71.4% of recovery with grade 3 was observed in sheep receiving intermittent vibration at 7-day interval; on the contrary, only 14.3% of recovery with grade 3 was found in sheep undergoing natural healing without vibration treatment).

In general, healthy bone should be high in mineral density and in bone volume fraction; and low in trabecular bone separation and in the number of trabecular bones with huge thickness. Once a healthy bone is injured (such as sham operation, internal fixation or fractures), bone mineral density and volume fraction decrease; furthermore, the number of trabecular bone increases, the thickness decreases and the degree of separation becomes larger^18^. Our results showed that, firstly, all measured 5 parameters (BMD, BV/TV, Th-N, Tb-Th, and Tb-Sp) by micro-CT showed no differences among sheep receiving vibration at different intervals, even though their grades of clinical recovery were very different. The control group had the highest BMD and Tb-Th, while other sheep that received surgery showed decreased BMD and Tb-Th. Secondly, Tb-N and Tb-Sp were higher in all sheep that received surgery than in those that did not. Thirdly, BV/TV was comparable in the control group, the sham control group and the internal fixation group. A recent study has shown that a low vibration treatment for rib fracture healing in rats only improved BV, and did not improve BV/TV, BMD, Tb-N, Tb-Th and Tb-Sp^19^. Those results suggested that there might be a complex mechanism between micro- CT measured parameters and vibration treatment for bone fracture healing.

For the control group, the sham control group and the internal fixation group, there were similar change patterns of BMD, BV/TV, Tb-N and Tb-Th; *that is,* there were no significant differences in BMD, BV, Tb-N and Tb-Th between the sham control group and the internal fixation groups; for which we are unable to explain right now. BMD and BV/TV were significantly increased in the internal fixation group, which might be explained because the stress occlusion of steel plate influenced bone reconstruction^1^ ^,^ ^3^.

We especially noticed that no apparent differences of 5 measured microstructure parameters and of E values of elastic modulus could be observed between sheep undergoing natural healing and those receiving vibration (Figs. 3–4, Table 2). The results do not seem to be completely consistent with the results of fracture healing grade observed, in which the healing grade was higher in sheep receiving intermittent vibration at 7- or 5-day interval than in the natural healing group (71.4% in sheep receiving intermittent vibration at 7-day interval and 57.1% in sheep receiving intermittent vibration at 5-day interval had grade 3 healing; however, 71.4% of sheep undergoing natural healing had grade 2 healing). We speculated that it might be due to the limited sample size.

Moreover, we also noticed that all parameters of the internal fixation group showed a very different characteristic compared with sheep receiving vibration. No differences of those microstructure parameters were observed between sheep receiving continuous vibration and those receiving intermittent vibration, and also among sheep receiving intermittent vibration at different intervals.

## Conclusion

Clinical healing effects should be the top concern. The quantitative analyses of bone microstructure and of finite element mechanics on the process of fracture healing need to be further investigated.
